# Trends in hospitalization for paediatric flatfoot: an Italian nationwide study from 2001 to 2016

**DOI:** 10.1186/s12887-022-03145-0

**Published:** 2022-02-08

**Authors:** Umile Giuseppe Longo, Rocco Papalia, Sergio De Salvatore, Laura Ruzzini, Vincenzo Candela, Ilaria Piergentili, Leonardo Oggiano, Pier Francesco Costici, Vincenzo Denaro

**Affiliations:** 1 Department of Orthopedic and Trauma Surgery, Campus Bio-Medico University of Rome, Rome, Italy; 2 Research Unit of Orthopaedic and Trauma Surgery, Fondazione Policlinico Universitario Campus Bio-Medico, Via Alvaro del Portillo, 200 - 00128 Roma, Italy; 3 Research Unit of Orthopaedic and Trauma Surgery, Department of Medicine and Surgery, Università Campus Bio-Medico di Roma, Via Alvaro del Portillo, 21 - 00128 Roma, Italy; 4 Department of Surgery, Orthopedic Unit, Bambino Gesù Children’s Hospital, Rome, Italy

**Keywords:** Flatfoot, Pes planus, Flatfeet, Arthroereisis, Subtalar joint fusion, Epidemiology

## Abstract

**Background:**

Flatfoot is a common condition in young patients, but usually resolves by adolescence. This study aimed to estimate annual trend hospitalizations for flatfoot in Italian paediatric population from 2001 to 2016.

**Methods:**

Data of this study were collected from the National Hospital Discharge Reports (SDO) reported at the Italian Ministry of Health regarding the years of this paper (2001–2016). The yearly number of hospital admission for flatfoot, the percentage of males and females, the average age, the average days of hospitalization, primary diagnoses and primary procedures in the whole Italian population were calculated using descriptive statistical analyses.

**Results:**

109,300 hospitalizations for flatfoot of young patients were performed during this period. 59.3% of patients were male and 40.7% female of the 10–14 years-old age class. The average days of hospitalization stay were 1.73 ± 1.27 days. The data highlights that the burden of flatfoot surgery is growing and affecting the healthcare system. The mean rate of hospital admissions in Italy for flatfoot in the young population was 82.14 for 100,000 inhabitants of the same age class.

**Conclusions:**

The data highlights that the cases of flatfoot surgery increased from 2001 to 2016. The most common treatment was the “Internal Fixation Of Bone Without Fracture Reduction, Tarsals And Metatarsals followed by Subtalar Fusion and Arthroereisis. Further prospective studies on this topic may be conducted to improve the evidence of the results.

## Background

Pes planus (flatfoot) is one of the most common benign conditions affecting the pediatric population, and it is generally solved during adolescence [1]. By definition, flatfoot has a decreased or absent longitudinal medial arch. The deformity may be the only sign or may be associated with other abnormalities as valgus alignment. Paediatric flatfoot can be divided into flexible (with or without Achilles tightness) and rigid forms, characterized by pathological reduction of subtalar joint range of motion [2, 3]. According to aetiology, it is possible to distinguish between Congenital (CF) and Acquired forms (AF) [4]. Epidemiological studies have demonstrated that pes planus is the physiological shape of the foot in the first years of life [1]. Morley and colleagues [5] reported a 97% prevalence of pes planus (estimated by the heel-to-arch width ratio) in patients under six years old. The medial longitudinal arch tends to develop between three and six years [6]. The prevalence of this condition decreases during the growth, reaching 4% of prevalence at ten years [6]. Chen et al. [7] found that male gender, obesity, severe joint laxity and W-sitting are related to an increased risk of suffering from symptomatic pes planus. The presence of flatfoot until ten years old is physiological [8] because it is often flexible, without functional limitations and asymptomatic. In limited cases, pes planus could become symptomatic. There is no international consensus on the proper management of this condition (either surgical or conservative), and opinions differ between countries [9]. However, in most cases of painless flexible flatfoot, surgery is not required. On the contrary, orthotics or surgical intervention may be considered if the patient is symptomatic. Unfortunately, there are insufficient high-quality studies to prove the efficacy of orthotics for flatfoot [10]. Pfeiffer and colleagues [11] suggested that more than 90% of the orthotics treatment are unnecessary. On the other hand, surgical therapy is required for symptomatic instances that do not respond to conservative treatments and rigid forms. Even if numerous researches about pes planus aetiology [1, 2, 4] and treatments [10] were published, few information on hospital admissions trends are found, and no public database or registry on this population was available. National health statistics for flatfoot could be interesting for an international audience, as different surgical management is described between countries (e.g. mean age at the time of surgery, type of surgical intervention). These distinctions enable worldwide comparisons of outcomes. Furthermore, providing national statistics and connecting them with protocols from other nations might help compare outcomes, giving the possibility to establish an international consensus on the best management of this condition.

The purpose of this study was to determine the annual number of hospital admissions for flatfoot in children (0–14 years old) in Italy between 2001 and 2016. All the data were harvested from the National Hospital Discharge Reports (SDO).

## Methods

The data for this study came from the SDO reports filed with the Italian Ministry of Health. All information relates to the period between 2001 and 2016. The SDO publishes all data on each surgical treatment done in Italy. The information in the SDO relates to the patient's characteristics (age, sex, length of hospital stay, diagnosis and procedure performed). The Italian National Institute of Statistics (ISTAT) in Italy publishes national and regional population data every year. Paediatric flatfoot was determined by the International Classification of Diseases, Ninth Revision, Clinical Modification (ICD-9-CM) major primary diagnosis code 734 (Acquired flatfoot) and 754.61 (Congenital pes planus). The analysis of flatfoot was conducted in patients under 15 years old. Authors defined as "young" a patient aged between 0–14 years (according to ISTAT [12, 13]). The principal included surgical procedure codes were: 78.58 (Internal Fixation Of Bone Without Fracture Reduction, Tarsals And Metatarsals), 81.13 (Subtalar Fusion), 78.48 (Other Repair or Plastic Operations on Bone, Tarsals And Metatarsals) and 81.18 (Subtalar Joint Arthroereisis).

In Italy, the healthcare system is based on the Beveridge model, in which the government is responsible for public health. The combination of public and private healthcare is known as private healthcare. The S.D.O. gathers data on the types of reimbursements (public or private) [14, 15].

Descriptive statistical analyses were performed. As appropriate, continuous and categorical variables were summarized as the mean and standard deviation or the count and percentage. The Kolmogorov–Smirnov and Shapiro–Wilk test were performed to assess the normality distribution. The Mann–Whitney U test assessed the statistically significant differences between males and females in age and length of hospitalization. The statistically significant differences between age groups in the length of hospitalization with the Kruskal–Wallis test were assessed. The pairwise comparisons were assessed using the Mann–Whitney U test with Bonferroni correction. Categorical data were compared with the Chi-square test. The yearly number of youths aged 0 to 14 years old retrieved from ISTAT, a national electronic registry of the community, was used to determine the incidence. All statistical analyses with SPSS version 26 (Armonk, NY: IBM Corp) and Microsoft Excel (2019) were performed.

## Results

### Population

**Fig. 1:**
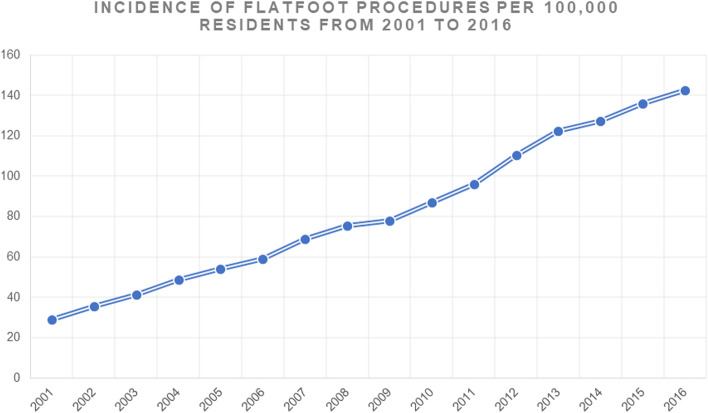
Incidence of flatfoot procedures × 100,000 resident from 2001 to 2016

**Fig. 2:**
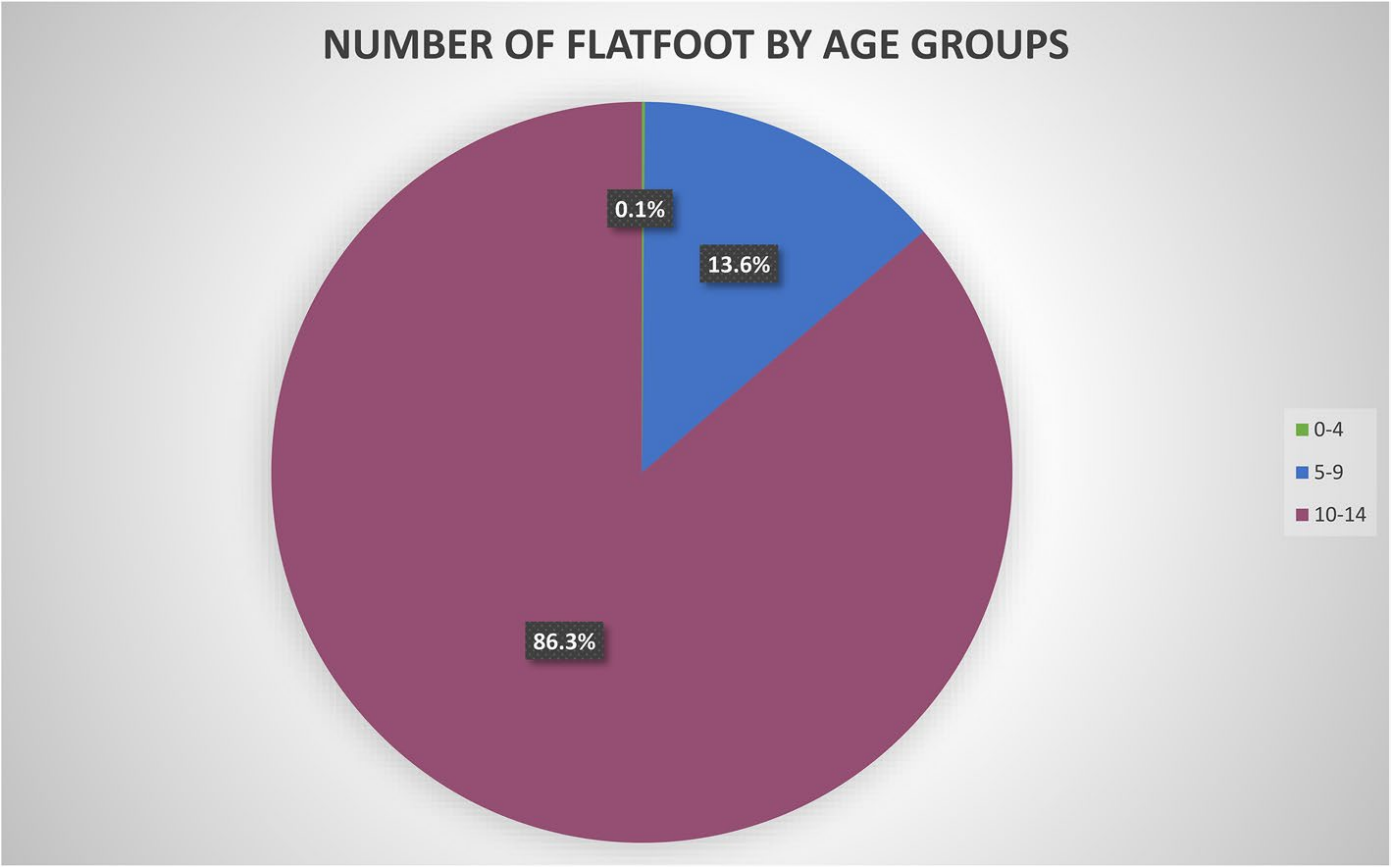
Number of flatfoot procedures by age groups

Between 2001 and 2016, 109,300 admissions for flatfoot were performed in Italy. From 2001 to 2016, the prevalence of hospitalization of young people for treatment of symptomatic flatfoot increased from 2349 in 2001, with the peak reached with 11,684 in 2016. The cumulative incidence period was 82.14 flatfoots for every 100,000 Italian young inhabitants. Between 2001 and 2016, the incidence increased from 28.97 in 2001 to 142.79 in 2016 (Fig. 1). The 10–14-year-old age group had the most significant percentage of operations performed (Fig. 2). The mean age was 11.23 ± 1.63 (males: 11.3 ± 1.7 years and females: 11.2 ± 1.6 years; *p* < 0.001). The increase in mean age from 11.01 ± 1.90 in 2001 to 11.46 ± 1.51 in 2016 was found. Men represented the majority of patients treated for flatfoot, 59.3% of males and 40.7% of females. During the 16-years period, the percentage of males is always higher than that of females (*p* = 0.012). From 2001 to 2016, the M/F ratio decreased from 1.56 to 1.46 during 15 years of study.

### Length of the hospitalization

**Fig. 3:**
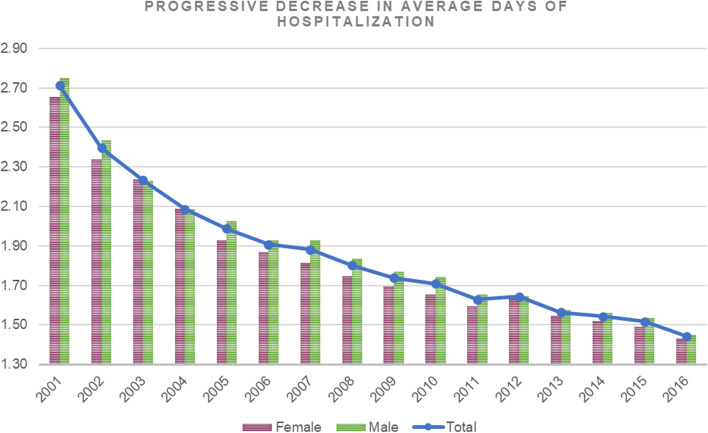
Progressive decrease in average days of hospitalization

The average days of hospitalization stay were 1.73 ± 1.27 days (males: 1.8 ± 1.3 days and females: 1.7 ± 1.3 days; *p* < 0.001). A decrease in mean hospitalization stays from 2.71 ± 1.83 days in 2001 (2.65 ± 1.65 days for females and 2.75 ± 1.94 days for males) to 1.44 ± 0.86 days in 2016 (1.43 ± 0.81 days for females and 1.45 ± 0.89 days for males) was found (Fig. 3). The average days of hospitalization in the age group 0–4 was 2.8 ± 2 days, in the age group 5–9 was 1.8 ± 1.3 days and in the 10–14 age group was 1.7 ± 1.3 (*p* < 0.001). All the pairwise comparisons were statistically significant different (*p* < 0.001).

### Diagnosis and procedure codes

**Fig. 4:**
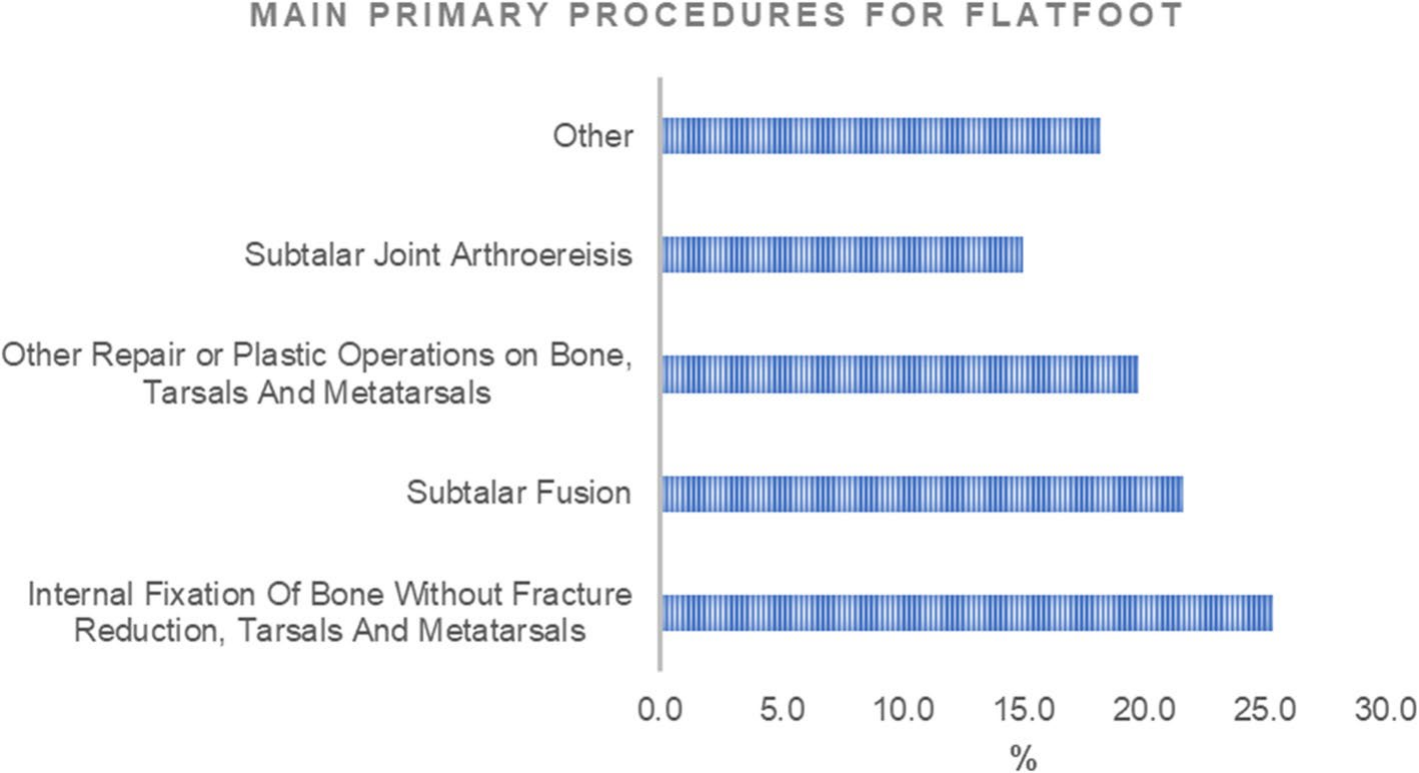
Main primary procedures for flatfoot

**Fig. 5:**
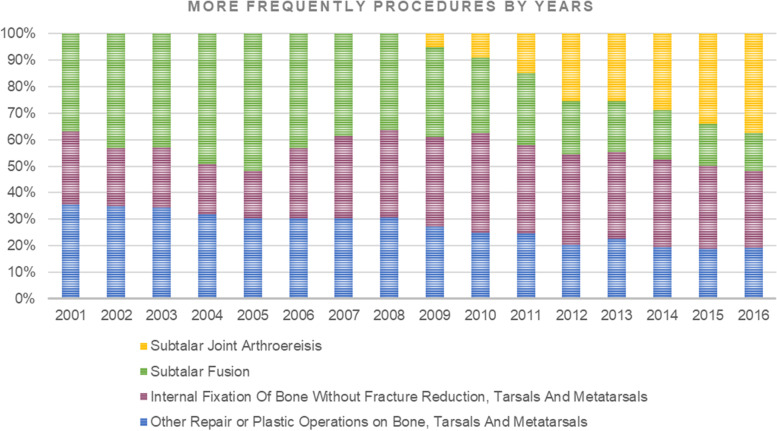
Most frequently performed procedures by years

The main primary admission diagnosis code consisted of the 754.61 (Congenital pes planus) with 56.1% of prevalence and 734 code (Flat foot) with 43%. The major primary procedures codes were 78.58 (Internal Fixation Of Bone Without Fracture Reduction, Tarsals And Metatarsals; 25.3%), 81.13 (Subtalar Fusion; 21.6%), 78.48 (Other Repair or Plastic Operations on Bone, Tarsals And Metatarsals; 19.8%) and 81.18 (Subtalar Joint Arthroereisis; 15%), (Fig. 4). From 2001 to 2008, the major procedure performed was Subtalar Fusion (code 81.13), while, from 2009 to 2016 the most procedure performed was Subtalar Joint Arthroereisis (code 81.18) (Fig. 5).

## Discussion

Pes planus is a common condition in the young population and is frequently encountered in adults [16]. From 2001 to 2016, the rate of hospital admissions in Italy for flatfoot patients under 15 years old increased from 28.97 in 2001 to 142.79 (× 100,000 inhabitants). The data harvested from the SDO reported that the most common treatment was the "Internal Fixation Of Bone Without Fracture Reduction, Tarsals And Metatarsals followed by Subtalar Fusion and Arthroereisis (Fig. 5). Pes planus could be divided clinically into flexible or rigid forms and etiologically in congenital or acquired forms [17]. "Flexible flatfoot" does not have a proper code in ICD-9-CM. As other conditions of flatfoot involve a wide range of topics, the aim of this discussion is focused on the flexible form of flatfoot that constitutes the most common and benign condition [18]. Most hospitalizations to treat flatfoot were performed in subjects from 10–14 years old. In this age class, young patients increase their sports activity, leading to an increment in symptomatic forms. Concerning the days of hospitalization, a progressive decrease during the years was found.

Most cases of flexible flatfoot do not need surgical intervention [19–21]. The presence of symptoms is the essential consideration in determining whether to treat conservatively or surgically [22–24]. Pain, fatigue of the foot muscles, quick and frequent shoe breakdown, ankle sprains, and calluses on the medial portion of the foot are some of the symptoms [25]. Many instances are asymptomatic, and non-invasive treatments are frequently used (casting, orthoses and modified weight-bearing). Otherwise, there is no high-quality evidence that orthotics or surgery can reduce the odds of future problems. The use of orthotics in symptomatic form is contentious [26, 27] and there is currently insufficient data to support this approach. Only a few non-comparative studies assessed the advantages of orthotics in patients with flexible flatfoot [10, 28]. On the other hand, surgery may be useful in children who are in discomfort, with or without a short Achilles tendon [29–31]. Soft tissue plications, osseous excisions, osteotomies (medial cuneiform osteotomy, medial slide calcaneal osteotomy, lateral column lengthening) [32–34], tendon lengthening or transfers, arthroereisis, and combinations of these procedures could be used to treat flexible flatfoot caused by constitutional laxity or other acquired conditions (trauma, obesity, [35–38] tumors, infections, neurological diseases). The most interesting finding of this study was related to the progressive rising trend in operative management for acquired flatfoot. The most frequent surgery performed is arthroereisis of the subtalar joint [39, 40], which is minimally invasive and carries low surgical risks [41, 42]. This data could probably constitute the most important outlier of the study. In fact, the recent increase in the trend of subtalar arthroereisis could have different reasons. The parents often perceive subtalar arthroereisis as “low risk” with a broader acceptance of surgery. However, no data regarding the reason for this increase could be obtained through the ICD-9-CM code; therefore, it is not possible to report a significant conclusion on this topic. Bernasconi et al. [41] performed a systematic review on the use of arthroereisis to treat pediatric flatfoot. They reported a rate of complication that ranged from 0 to 11%. However, the American Orthopaedic Foot and Ankle Society reported 33% of complications (mainly implant mobilization) [43]. Moreover, the review by Shah and colleagues [43] reported the comparison between the trend to perform arthroereisis in non-United States- countries and the United States- countries. Authors sustained that the type of intervention could be influenced by the healthcare system payment [43]. Further studies are required to assess the reasons for the progressive trend in pediatric flatfoot surgeries.

Our study has some limitations. The ICD-9-CM classification for all the procedures reported was used. However, various codes for the same surgical procedure might be used with the ICD-9-CM system. Indeed, different codes were used in SDO to record subtalar joint arthroereisis (i.e. subtalar joint arthroereisis, subtalar joint fusion and internal fixation of bone without fracture reduction, tarsals and metatarsals). The code "Subtalar fusion" was also used when absorbable screws were used for subtalar joint arthroereisis. A limitation of the ICD-9-CM code is that there is no specific ICD-9-CM code for the flexible flatfoot. Therefore, a flexible flatfoot is reported in the ICD-9-CM System either as “acquired” or “congenital forms”. This heterogeneity of codes may generate mistakes in data reporting and interpretation. Future endeavours should be focused on the differentiation between the two diagnoses.

## Conclusions

The data highlights that the burden of flatfoot surgery is growing and affecting the healthcare system. The mean rate of hospital admissions in Italy for flatfoot in the young population increased from 28.97 in 2001 to 142.79 (× 100,000 inhabitants). The most common treatment was the Internal Fixation Of Bone Without Fracture Reduction, Tarsals And Metatarsals followed by Subtalar Fusion and Arthroereisis. Further prospective studies on this topic may be conducted to improve the evidence of the results.

## Data Availability

The datasets used and/or analyzed during the current study are available from the corresponding author on reasonable request. The access to the database is on request. All data were obtained by the Direzione Generale della Programmazione Sanitaria—Banca Dati SDO of the Italian Ministry of Health.

## Abbreviations

AF: Acquired forms
CF: Congenital forms
ICD-9-CM: International Classification of Diseases, Ninth Revision, Clinical Modification
SDO: National Hospital Discharge Reports
ISTAT: Italian National Institute of Statistics
